# Dyskinesias in childhood, differential diagnosis and treatment. About a case

**DOI:** 10.1192/j.eurpsy.2022.1116

**Published:** 2022-09-01

**Authors:** M. Valverde Barea, C. Mata Castro, P. Vargas Melero, A. España Osuna

**Affiliations:** 1 Universitary Hospital of Jaén, Psychiatric, Jaén, Spain; 2 Universitary Hospital of Jaén, Psychiatric, Jaen, Spain

**Keywords:** Dyskinesias, stereotypies, autism, Antipsychotics

## Abstract

**Introduction:**

Dyskinesias are motor disorders that occur as a side effect to treatment with typical and less frequently with atypical antipsychotic drugs. They are more frequents in child population. Treatment usually consists of decrease the dose of drug or replace it with a better profile tolerability antipsychotic. Clozapine is an antipsychotic drug indicated as second-generation treatment of motor disorders that appear as side effects to treatment with neuroleptics.

**Objectives:**

Demonstrate the efficacy and tolerability of clozapine in the treatment of dyskinesias in childhood.

**Methods:**

The patient 12 year-old boy, has episodes of psychomotor agitation once a month. This will alternate with quiet moments in which dyskinetic movements are observed in upper limbs, without being able to detect any type triggering environmental factor. Personal history: hydrocele, diagnosed at 8 years becomes neurodevelopmental disorder considered. Neurosurgery tracking for Subarachnoid cyst. Psychopathological examination: Child presents psychomotor restlessness, disruptive behavior, impairments in communication, movement disorder, stereotypies and dyskinetic movements in shoulder and neck.

**Results:**

In the patient suffering from an autistic disorder, stereotypies and other motor symptoms were observed, the predominant and most relevant being dyskinetic movements in the shoulder and neck, which appeared one month after starting treatment with risperidone and worsening psychomotor skills. Treatment of dyskinesia with clozapine improved the motor symptoms presented by the patient.

**Conclusions:**

Clozapine should be the treatment of choice in the event of dyskinesias as a secondary effect to other antipsychotic treatments, proving effective in controlling them as well as well tolerated in both adults and children.

**Disclosure:**

No significant relationships.

